# 

**Published:** 1915-03

**Authors:** 


CONTENTS.

ORIGINAL COMMUNICATIONS—

A Trip Through Europe and the Hospitals, by L. C. Fischer, M. D. ....555

Meeting of the Fulton County Medical Society, by R. R. Daly, M. D. ....567

EDITORIAL ....578

SELECTIONS AND ABSTRACTS ....582

BOOK REVIEWS ....608

